# A Case of Locally Advanced Fibrosarcoma in a Young Male

**DOI:** 10.7759/cureus.44095

**Published:** 2023-08-25

**Authors:** Ali Raza, Imrana Siraj, Sabina Malik, Raji Mohammed, Masood A Shariff

**Affiliations:** 1 Surgical Oncology, New York City (NYC) Health and Hospitals Corporation (HHC) Lincoln, Bronx, USA; 2 Cancer Center Research Services, New York City (NYC) Health and Hospitals Corporation (HHC) Lincoln, Bronx, USA; 3 Cancer Center, New York City (NYC) Health and Hospitals Corporation (HHC) Lincoln, Bronx, USA; 4 Pathology and Laboratory Medicine, New York City (NYC) Health and Hospitals Corporation (HHC) Lincoln, Bronx, USA

**Keywords:** high-grade, limb amputation, surgical oncology, sarcoma, fibrosarcoma

## Abstract

Fibrosarcoma is a rare, highly malignant tumor that develops from uncontrolled overgrowth of fibroblastic cells. It may start as a painless lump or swelling under the skin. But as it grows, it can push surrounding structures - organs, muscles, nerves, or blood vessels - and lead to pain and tenderness. The treatment of fibrosarcoma depends on several factors such as size, grade, location of primary tumor, extent of spread, age, and general health condition of the patient. The main treatment is surgical removal of the primary tumor with wide-margin excision and amputation if localized in the limb. Prior to surgical intervention, radiotherapy can be applied to reduce the tumor size or following surgery to lower the risk of recurrence. Chemotherapy is indicated in cases of metastasis. Unfortunately, the prognosis of fibrosarcoma is not favorable. For high-grade fibrosarcoma, the five-year survival rate is around 30% and for low-grade fibrosarcoma, it is 50-80%, with recurrence in the first two to five years post-surgery. We encountered a case of high-grade fibrosarcoma with aggressive growth in a 36-year-old male, requiring above-knee amputation.

## Introduction

Fibrosarcoma is a fibroblastic neoplasm originating from mesenchymal cells, also known as soft-tissue sarcoma that is part of a broad heterogeneous group of tumors that comprise more than 175 molecular subtypes [1]. It affects people of all ages, but a higher incidence is noted in the third to sixth decades of life, predominantly in men [1,2] and frequently seen near the skin surface, tendons, and fascia of the deep soft tissue to within the periphery of bones. Many subtypes with varying clinical manifestations based on size, location, and pressure effect in nearby tissues ranging from non-specific soft tissue mass to primary or secondary bone tumor [3,4].

Benign and malignant neoplasms mimic fibrosarcoma. Low-grade fibromyxoid sarcoma, malignant fibrous histiocytoma, sclerosing epithelioid fibrosarcoma, dermatofibrosarcoma protuberans, extra-abdominal desmoid fibromatosis, leiomyosarcoma, liposarcoma, and synovial sarcomas are some of the differentials of true adult fibrosarcoma. Cytogenetic analysis can be used to detect and characterize chromosomal abnormalities that help in diagnosis [1,4]. The molecular changes in fibrosarcoma are not consistent and findings vary with heterogeneous diseases [1]. Some of the most common changes that occur are in the form of loss of chromosomal genes like 13q (which results in mutation in the RB1 gene (retinoblastoma gene)). Also, other genes whose functionality is lost are, for example, 10q, which is a tumor suppressor gene, and some are gained like 17p (TP53, tumor regulator protein) [2,4]. These chromosomal changes could act as future therapeutics for targeting and downregulation of the expression of genes to prevent recurrence. Desmoid tumors are aggressive fibromatosis that does not metastasize but instead exhibit local invasiveness; surgical excision combined with radiation therapy is performed, with systemic chemotherapy offered due to the sporadic nature of the tumor [5]. Dermatofibrosarcoma depicts mutation in the CTNNB1 gene, which encodes beta-catenin and is associated with the beta-catenin pathway and has a female preponderance, especially following pregnancy arising from the abdominal wall.

Imaging tests can be done to identify the detailed picture of the tumor and confirmation with biopsy and staining to distinguish between fibrosarcoma and other spindle-cell neoplasms with the use of immunochemistry is considered though it is not sufficient. New markers, such as miRNA expression profiles, may represent an additional supportive diagnostic step [6]. If the location of the tumor is in a limb, some bone may need to be removed followed by replacement with a prosthesis or bone graft. Amputation may be necessary when the tumor involves vascular structures, in some cases [7,8]. Although there is no definitive cause, the risk of fibrosarcoma increases in patients with preexisting trauma to bone or soft tissue, postradiotherapy, or in rare instances, implantation of foreign material [9,10]. Increased incidence of fibrosarcoma is linked to bone infraction, chronic osteomyelitis, fibrous dysplasia, Paget disease, as well as, neurofibroma (10% lifetime risk) [1,2]. There are many tumors in the differential diagnosis, including spindle cell melanoma, spindle cell squamous cell carcinoma, synovial sarcoma, leiomyosarcoma, malignant peripheral nerve sheath tumor, and biphenotypic sinonasal sarcoma, but immunohistochemistry with positive vimentin stain with negative muscular immunomarkers aids in diagnosis. Histologically, fascicles of spindle-shaped cells resemble a “herringbone pattern” [9-11]. Fibrosarcoma often metastasizes to the lungs, bone of the axial skeleton, and, rarely, to lymph nodes with a five-year survival rate of 39-54% [9,11]. The current therapy of choice is radical surgery; radiation can be used as adjuvant in inoperable cases or chemotherapy as palliative treatment [2,11]. Prognosis is directly related to histological grade, tumor size, and adequate surgery (margins free of tumor) [2,12].

We report a case of high-grade fibrosarcoma (30 cm in size) in a 36-year-old man, originating in the thigh and requiring surgical above-knee amputation.

## Case presentation

**Figure 1:**
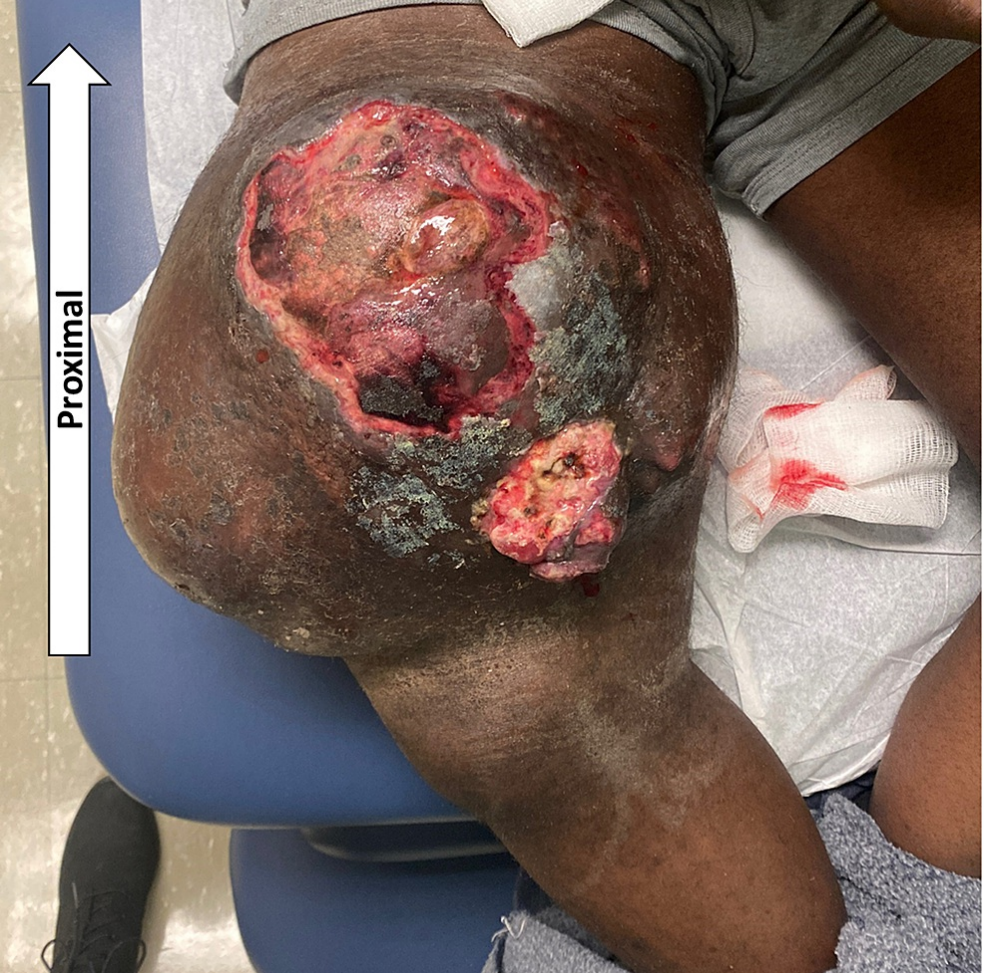
The gross image of the tumor on the right anterior thigh, with draining sites (arrow)

A 36-year-old black male presented with the chief complaint of draining blood and pus from a mass in the right thigh that, as per the patient, had been present for approximately seven years and had been enlarging steadily over time and recently started draining. He hadn’t reached out for any medical assistance before this. The patient had difficulty bending the right knee and mentioned the occasional loss of strength in the affected extremity and falling down. On examination, the mass was 30 cm by 25 cm and was firmly fixed in the anterior thigh, and abutting the underlying musculature. Areas of denudation, ulceration, and exophytic areas with punctate bleeding and serous discharge were noted (Figure 1). Additionally, the patient was deconditioned with fatigue on walking short distances without complaints of shortness of breath, as he had limited his activity over time and started staying home because the mass had limited his ambulation and became a hindrance. The patient denied pain and weight loss, and on examination, did not have orthostatic hypotension. On examination, the right lower limb had a limited range of motion at the knee joint and associated inguinal lymphadenopathy.

**Figure 2:**
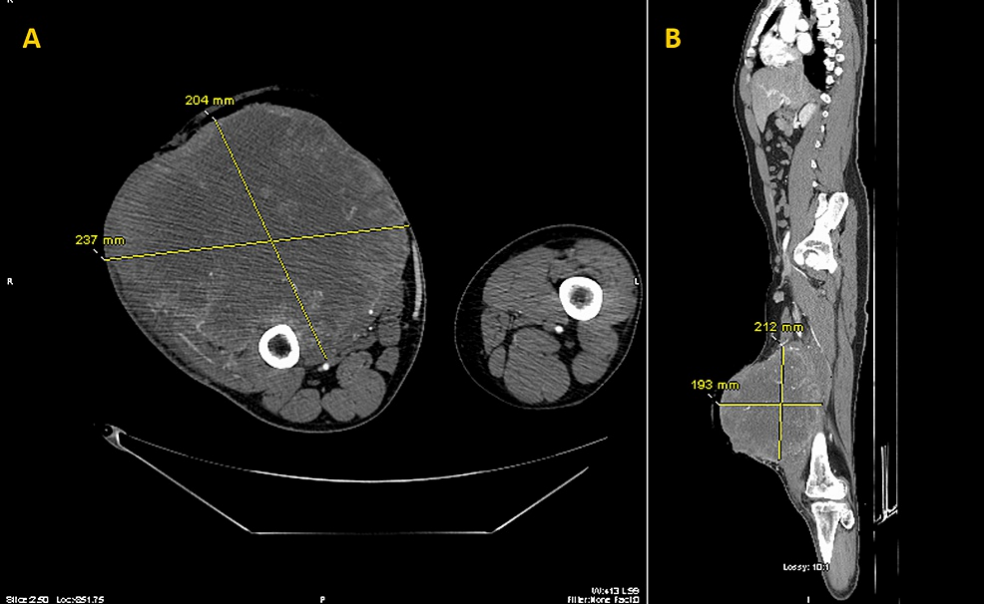
CT image of the right lower limb with fibrosarcoma protruding anteriorly from the quadriceps muscle A - Axial Plane, B - Sagittal Plane

At presentation, CT angiography of the right lower extremity was done, which showed a 22 cm necrotic mass in the anterior compartment of the right leg at the level of the distal femur, which had replaced nearly all of the anterior thigh musculature with no erosion of the adjacent femoral diaphysis (Figure 2). CT-angiogram of the lower extremity noted multiple irregular arteries throughout the mass arising from the deep femoral artery. Radiologically, the mass appeared to extend to the skin whilst displacing the adductor muscular compartment posteriorly, avoiding neurovascular involvement. The mass capsule did not react with the femur and no cortical erosions were noted. Right inguinal adenopathy (measuring 4.4x1.6 cm) presumably metastatic was noted on the scan. Additionally, a Doppler ultrasound of the right lower extremity revealed sluggish flow in the popliteal vein.

**Figure 3:**
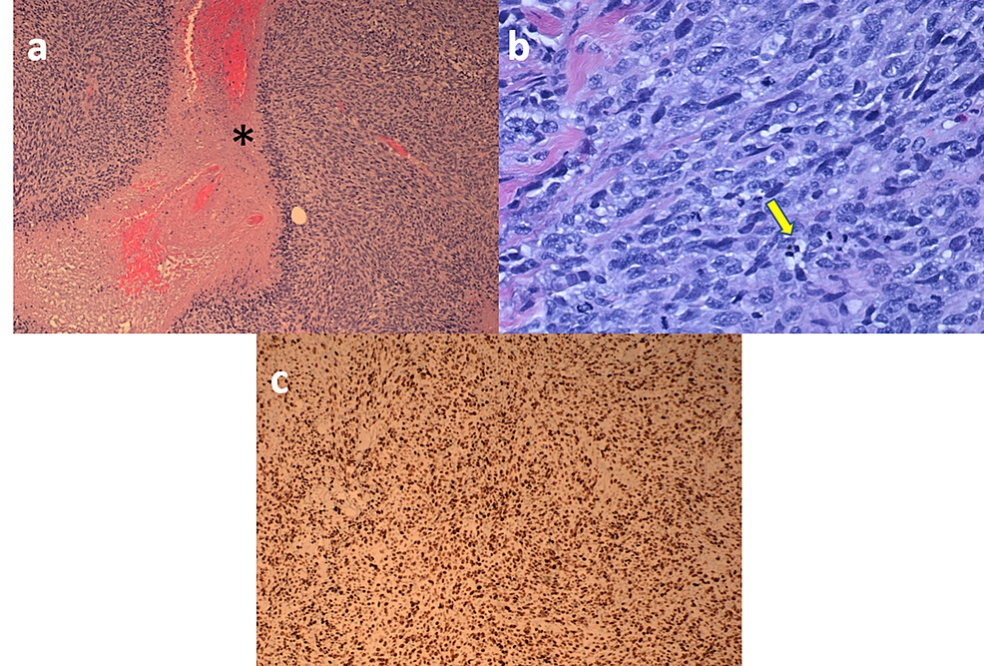
(a) Markedly hypercellular spindle cell sarcoma with prominent necrosis (*) (H&E stain, original magnification X100). (b) Hypercellular spindle cell sarcoma with brisk mitotic activity (arrow) (H&E stain, original magnification X400). (c) Ki-67 immunostaining with a high proliferation index (70-80%).

Excisional biopsies of the mass and inguinal lymph nodes were performed and revealed high-grade spindle cell sarcoma, consistent with fibrosarcoma and benign lymph nodes with marked follicular lymphoid hyperplasia and sinus histiocytosis (Figure 3). The drainage at presentation was due to tumor necrosis that had developed due to a very high-grade presentation of the sarcoma and was staged at T4NoMx.

The case was discussed at a multidisciplinary meeting and due to the advanced nature of the fibrosarcoma, an above-knee amputation (AKA) was recommended, followed by radiation. The orthopedic prosthetic specialists were consulted regarding femur preservation and guidance to the above knee amputation was recommended with guidance to permit prosthetic assignment. Regarding a transfemoral amputation, 50% of the femoral bone needs to be preserved with myodesis. In our case, the complete length of the hamstring would be excised, depending on clear margins. The quadriceps wound is preserved and brought forward. The stump would be fashioned for future prostheses. Debulking of the quadriceps was an alternative, leaving the patient with a nonfunctional limb and an increased risk of positive margins.

The patient returned to the emergency department four days post-biopsy. On examination, seropurulent discharge was present with an associated leukocytosis of 17.79/uL, and sepsis was suspected the patient was started on empiric antibiotics and admitted to the ICU. An amputation was agreed upon as the definitive procedure. A week later, a high amputation was performed with the anterior incision approximately 4 cm above the gross tumor and closure with a posterior flap. The hospital course was complicated by anemia for which a blood transfusion was given. There was gradual resolution of the leukocytosis postoperatively with stabilization of his hemoglobin level prior to discharge. One week after surgery, the patient was admitted to acute inpatient rehabilitation, received physical and occupational therapy services, and made steady progress in performing Activities of Daily Living (ADL) and functional mobility. The patient was provided with a wheelchair and skilled training to improve safety during functional transfers and ADLs. The patient was transferred to a skilled nursing facility for wound management and physical therapy, with an eventual recommendation for shaping the residual limb and prosthesis fitting.

## Discussion

Our case presentation highlights a young man diagnosed with an aggressively growing fibrosarcoma in his right anterior thigh involving the entirety of the quadriceps muscle. This patient represents a population in the South Bronx, a region that is a low-socioeconomic sector of New York City. Access to care is affected by many factors, as in our patient's case, where he did not seek early medical attention due to lack of insurance. Possible un-education limited this patient's suspicion to a much more malign pathology. Cancer health disparities have grown as per the National Cancer Institute [13], and efforts must be in place to increase awareness for a wide variety of disparities. The aggressive nature of the disease, lack of wound care following the incisional biopsy, and newer eruptions in the region of the thigh led to a septic presentation followed by amputation.

The morbidity of this young patient brings to light a possible surveillance mechanism for patients and primary care physicians when they encounter a mass. Urgent referral to a surgeon and an oncologist should be the priority. Fibrosarcoma mainly arises in areas of collagen-rich connective tissue, most commonly around the thighs, knees, arms, and trunk. Fibrosarcomas are usually spherical masses of firm consistency with sharp demarcation from surrounding tissue and on average 3-8 cm in size [14]. Due to its deep location and painless nature, the tumor is often unrecognized for a prolonged period. Symptoms arise when the enlarging tumor compresses the surrounding tissue. Depending on the location of the tumor, pain from nerve impingement, disruption of blood supply, and restrictions in the movement of joints and major muscles present. Advanced stages of fibrosarcoma can result in anorexia, weight loss, and fatigue. Malignancy should be suspected if the size is greater than 5 cm, if it is situated in deep tissues, or if there is pain on presentation, and if there is continued growth of the tumor [15]. As necrotic centers form due to high-grade cell mitosis and outgrowth of its blood supply, these centers rupture the skin and drain fluid and pus with an increased risk of infection, as was the case with our patient.

In a clinical setting, there are more than 50 different histological types of soft tissue sarcomas, each with unique clinical manifestations and prognoses. Sarcomas are also different in terms of metastasis. Some sarcoma types tend to metastasize to regional lymph nodes such as epithelioid sarcoma [1-3]. Sarcoma heterogeneity can also be explained based on treatment response, as different patients or even different lesions in the same patient show different responses to the same treatment.

First, radiological imaging, such as MRI, is recommended, as it adequately demonstrates soft tissue structures. Histological characteristics typically exhibit monomorphic spindle-shaped fibroblasts arranged parallel to each other. Depending on the tumor size, lymph node involvement, and metastasis, fibrosarcoma is divided into the following stages - IA (low-grade, 5 cm or smaller); IB (low-grade, larger than 5 cm), IIA (mid-grade, somewhat faster grow/spread; or high-grade, has faster grow/spread and are 5 cm or smaller); IIB (mid-grade/high-grade are larger than 5 cm); III (high-grade, larger than 5 cm, and lymph node involvement); and IV (any grade, any size, lymph node involvement, and metastasized) [14]. Due to very similar morphology, tumor genetics and clinical manifestation are analogous in various spindle cell sarcomas with a likelihood of misdiagnosis. To differentiate fibrosarcoma from other spindle cell sarcomas, immunohistochemical and molecular techniques can be used. Surgical resection is the gold standard treatment. The type of surgery offered is subject to tumor extent. In the case of malignant features, radiation therapy is highly recommended after tumor resection [14].

Chemotherapy, such as doxorubicin, is recommended to reduce recurrence. Some other agents, such as actinomycin D and ifosfamide, are also effective, with a response rate of above 15%. Neoadjuvant treatment with the MAID (mesna, doxorubicin, ifosfamide, and dacarbazine) regimen has proven beneficial [16]. A theory of communication between tumor cells and their surrounding stromal tissue plays a crucial role in cancer progression, invasion, metastasis, and chemosensitivity. Therefore, controlling the microenvironment surrounding the tumor is thought to have a high therapeutic potential for controlling tumor growth and enhancing chemosensitivity. The outcome of deaths was due to the recurrence of sarcoma with metastatic disease.

Data from the National Cancer Institute's Cancer Surveillance, Epidemiology, and End-Result (SEER) program showed that blacks were more predisposed to the development of sarcomas [16]. The genomic alterations are different among various sarcomas. Soft tissue sarcomas can be divided into two broad genetic groups: those with simple karyotypes harboring specific genetic alterations and those with complex karyotypes. The first group of diseases often presents with recurrent chromosome translocations, resulting in the fusion of genes and proteins. These fusion genes and proteins are mostly tumor-promoting [2,4]. On the other hand, the second group of diseases often presents with significant genomic instability and copy number alterations. Both gene copy number gain and loss are detected in these cancer types. Identifying the specific genomic alterations helps in the diagnosis and treatment plan of certain tumor types.

Cytogenetically, congenital infantile fibrosarcoma is characterized by the majority of cases having a translocation between chromosomes 12 and 15 (notated as t[12;15][p13;q25]) that results in the formation of the fusion gene, ETV6-NTRK3, plus individual cases exhibiting trisomy for chromosomes 8, 11, 17, or 20. However, studies have indicated that genetic alterations and few familial outpouchings are reported for the development of soft tissue sarcomas. Genetic studies have found a chromosomal rearrangement in some fibrosarcoma [17]. Childhood treatments are dependent on the extent of the disease, i.e., size and location, and determining surgical options, such as limb salvage, rotationplasty, or amputation, with radiation and chemotherapy. Limited studies have shown a possible link between soft tissue sarcomas and the development of other types of cancer.

A decision as to whether a major amputation is appropriate should be made in a multidisciplinary meeting and in conjunction with the patient. Forequarter and hindquarter amputations have been reported, indicated after disease recurrence has occurred following a previous amputation [18]. Limb-sparing surgery takes on a completely different approach to surgical oncological outcomes with an assessment of excision margins that are to be achieved, local recurrence, re-excision, and disease-free survival [19]. Recurrence does occur with a higher rate in salvage compared to amputation, where a six-year follow-up noted a recurrence of 8% in salvage and 3% in amputees [20]; thus, achieving a clear margin is a challenge.

## Conclusions

Fibrosarcomas or fibrosarcomata are rare and debilitating tumors, especially when the extremities are involved, with the possibility of amputation or limb salvage surgery with chemoradiation. Recurrence is high with soft tissue tumors, which also leads to revision of surgery. Striking a balance in achieving appropriate tumor resection whilst preserving limb function remains the central challenge in the management of the extremity. Early implementation of surveillance by the primary care provider could aid in the appropriate evaluation of the mass with surgical oncology and oncological services, especially in underserved areas.
